# ﻿Re-assignment of *Gongrosira leptotricha*, a newly-recorded species in China, to Stephanosphaerinia clade (Chlamydomonadales, Chlorophyceae): insights from morphological and phylogenetic analyses

**DOI:** 10.3897/phytokeys.262.152528

**Published:** 2025-09-05

**Authors:** Qiumei Lan, Qingyu Dai, Huan Zhu, Guoxiang Liu, Benwen Liu

**Affiliations:** 1 Key Laboratory of Algal Biology, Institute of Hydrobiology, Chinese Academy of Sciences, Wuhan 430072, China Chinese Academy of Sciences Wuhan China; 2 University of Chinese Academy of Sciences, Beijing 100039, China University of Chinese Academy of Sciences Beijing China

**Keywords:** Chlorophyta, Chlamydomonadales, Stephanosphaerinia clade, *
Gongrosira
*, 18S rDNA, ITS, *tuf*A

## Abstract

A newly-recorded green algal species in China, identified as *Gongrosira leptotricha* Raineri, exhibits distinctive morphological characteristics. The thallus of this specimen is spherical or slightly irregular, calcified and firmly adheres to stones in running water. The prostrate part consists of loose, thin-walled tissue, with extended filaments being cylindrical and sparsely branched. The terminal sporangia are only slightly larger than the vegetative cells, with smaller cell dimensions and a higher length-to-width ratio. Biflagellate zoospores were formed within terminal sporangia. Each cell contains one or two pyrenoids. Ultrastructural observations revealed that the pyrenoids are traversed by thylakoid membranes and no plasmodesmata were observed between adjacent cells, confirming that *Gongrosira leptotricha* should be excluded from the order Chaetophorales. Phylogenetic analyses of 18S rDNA, ITS and *tuf*A sequences indicate that *Gongrosira leptotricha* belongs to the Stephanosphaerinia clade of Chlamydomonadales. Within this clade, *Gongrosira leptotricha* and *Gongrosira papuasica* forms a distinct, independent branch and cannot be assigned to any currently-known genus within Chlamydomonadales, but the taxonomic status of other species within the genus *Gongrosira*, including the type species, remains unresolved. Therefore, the name *Gongrosira leptotricha* has been retained. Further in-depth research, particularly focusing on the type species *Gongrosira sclerococcus*, is needed to refine the classification system and resolve the taxonomic uncertainties within this genus.

## ﻿Introduction

The genus *Gongrosira* Kützing, a widely distributed group of green algae, often forms greenish layers or cushion-like structures on substrates such as stones, gastropods, aquatic plants and other hard surfaces (Wehr et al. 2015). Current knowledge of this genus is primarily based on the morphological features of its species. Distinctive traits include cushion-like formations, often encrusted with calcium carbonate crystals and a filamentous system comprising both prostrate and erect uniseriate filaments. The prostrate system, which may be single or multilayered, varies from loose to pseudoparenchymatous and produces erect filaments with short, blunt-tipped branches. Cells are generally cylindrical or slightly inflated, sometimes with thick, lamellate walls and each contains a prominent parietal chloroplast and one to several pyrenoids (Printz 1964; Tupa 1974; Wehr et al. 2015; Guiry and Guiry 2025).

The genus, first established, based on *Gongrosira sclerococcus* Kützing (Kützing 1843), had its valid name confirmed by Silva (1952). Over time, the taxonomic concept of *Gongrosira* has shifted as new species were described and linked to various chaetophoralean algae (Printz 1964; Bourrelly 1972; Tupa 1974; Sarma 1986), including members of the Trentepohliaceae (Fritsch 1935; Smith 1950). Initially classified within the Trentepohliaceae by Fritsch (1935) and Smith (1950), the genus was later re-assigned to the Chaetophorales by Printz (1964) due to its branched growth pattern, cytological features such as cell wall and pyrenoid structure and the presence of zoospores with eyespots. This reclassification has gained widespread acceptance amongst subsequent phycologists (Hu and Wei 2006; Wehr et al. 2015; Guiry and Guiry 2025).

Currently, 24 species and two varieties are recognised (Guiry and Guiry 2025); however, many remain taxonomically uncertain due to overlapping morphological characteristics (Wehr et al. 2015). Despite the large number of described taxa, molecular data are available for only a few species, leaving many classifications unresolved. Until recently, *Gongrosira fluminensis* Fritsch and *Gongrosira burmanica* Skuja have been phylogenetically confirmed and reclassified under the Ulvales (Ulvophyceae) within different families (Liu et al. 2019a; Liu et al. 2024). Another important species, *Gongrosira leptotricha* (Rainieri and Chodat et al. 1926), previously undocumented in China, was collected during this study.

The primary objective of this research is to elucidate the phylogenetic relationships of *Gongrosira leptotricha* Raineri, a newly-recorded species in China, by integrating morphological observations with phylogenetic analyses of 18S rDNA, ITS and *tuf*A sequences, thereby providing new insights into the taxonomic complexities of the genus *Gongrosira*.

## ﻿Materials and methods

### ﻿Isolation and culture of algal strains

Samples of *Gongrosira leptotricha* Raineri were collected from the Chishui River in Guizhou Province, situated within the National Nature Reserve for Rare and Endemic Fishes in the upper reaches of the Yangtze River. The voucher specimen for this research has been archived in the Freshwater Algal Herbarium at the Institute of Hydrobiology (HBI), Chinese Academy of Sciences, while a living culture is deposited in the Freshwater Algae Culture Collection, Institute of Hydrobiology (FACHB), Chinese Academy of Sciences. The detailed information is listed in Table 1.

**Table 1.** Collection information and GenBank accession numbers newly obtained in this study.

Strain	Isolator	Date of collection	Locality	GenBank accession numbers		
				18S rDNA	tufA	ITS
*Gongrosira leptotricha*FACHB-3650	Q.M. Lan	March 2024	Guizhou province, China; on the stones freshwater 28°36'56.95"N, 105°46'50.28"E	PQ860775	PQ859866	PQ871574

Algal colonies adhering to stones were carefully extracted using tweezers under an Olympus SZX7 microscope (Olympus Corp., Tokyo, Japan), with samples either preserved in 4% formalin or maintained in a live state. Natural samples were rinsed with double-distilled water and then inoculated in culture dishes containing sterilised BBM medium (Bischoff and Bold 1963), solidified with 1.2% agar. They were exposed to a photon fluence rate of 15–35 μmol m^–2^ s^–1^ in a 14:10 h light:dark cycle at 20 °C. Upon the emergence of algal colonies, they were transferred to fresh medium to achieve a pure monoculture.

The algae in the late growth stage were transferred to a fresh solid culture medium and a suitable volume of liquid culture medium was added. The cells were then treated in the dark for approximately 48 hours to induce the release of zoospores. Morphological observations at each developmental stage were performed using an Olympus BX53 light microscope (Olympus Corp., Tokyo, Japan), which was equipped with differential interference contrast (DIC) and an oil immersion lens for image capture.

### ﻿DNA extraction, PCR amplification and sequencing

Cells in the exponential growth phase were collected for genomic DNA extraction and transmission electron microscopy (TEM). Morphological examination at each developmental stage was carried out using an Olympus BX53 light microscope (Olympus Corp., Tokyo, Japan), equipped with differential interference contrast (DIC) and an oil immersion lens for capturing images. Natural samples were pretreated with 10% hydrochloric acid prior to imaging.

Genomic DNA was extracted using the UE Multisource Genomic DNA Miniprep Kit (UElandy, Suzhou, China), following the manufacturer’s protocol. Approximately 15 mg of filaments were placed in a 2 ml centrifuge tube with 0.5 mm glass beads and 350 μl of phosphate-buffered saline (PBS, pH 7.0). Cell lysis was achieved using a fully automatic sample rapid grinder JXFSTPRP-24L (Jingxin, Shanghai, China) at a grinding frequency of 60 Hz.

For 18S rDNA sequence amplification, primers 18SR and 18SF (Marin et al. 2003) were employed under the following conditions: initial denaturation at 94 °C for 5 min, 32 cycles of 94 °C for 50 s, annealing at 55 °C for 50 s, extension at 72 °C for 90 s and final extension at 72 °C for 10 min. ITS sequence amplification utilised primers NS7 m and LR1850 (Bhattacharya et al. 1996), with conditions set at initial denaturation at 94 °C for 5 min, 32 cycles at 94 °C for 1 min, annealing at 55 °C for 1 min, extension at 72 °C for 2 min and final extension at 72 °C for 10 min. The *tuf*A gene was amplified using primers *tuf*AF and *tuf*AR (Famà et al. 2002), with an initial denaturation at 94 °C for 5 min, 32 cycles at 94 °C for 1 min, annealing at 52 °C for 1 min, extension at 72 °C for 2 min and final extension at 72 °C for 5 min. The amplified products of 18S rDNA, ITS and *tuf*A were sequenced by Wuhan Tianyi Huayu Gene Technology Co., Ltd (Wuhan, China). Sequence editing and assembly were performed using ContigExpress Project (Invitrogen, Grand Island, NY, USA) and the sequences have been deposited in GenBank (http://www.ncbi.nlm.nih.gov/).

For TEM preparation, algal samples were fixed in 2% glutaraldehyde and 0.05 M phosphate buffer at 5 °C for 2 hours, followed by post-fixation in 1% osmium tetroxide and 0.05 M phosphate buffer at 5 °C for 2 hours. Samples were then treated overnight at 5 °C in 1% uranyl acetate and methanol. After fixation, dehydration was carried out through an ethanol series and the samples were embedded in Spurr’s resin containing propylene oxide. Ultrathin sections were cut using a Leica UC7 ultramicrotome (Leica, Wetzlar, Germany) and stained with uranyl acetate and bismuth oxynitrate. The sections were examined using a Hitachi HT-7700 transmission electron microscope (Hitachi, Tokyo, Japan) at an acceleration voltage of 80 kV.

### ﻿Molecular phylogenetic analyses

Based on BLAST search results, potential phylogenetic relationships and a broader range of green algal species, sequences were retrieved from GenBank (http://www.ncbi.nlm.nih.gov/) for phylogenetic analysis, specifically according to sequence similarity and coverage. A total of 52 18S rDNA, 15 ITS and 19 *tuf*A sequences were downloaded. These sequences are mainly those that have high similarity to our sequences, are closely related or are representative within this taxonomic group. All sequences were initially aligned using MAFFT v.7.2 (Katoh and Standley 2013), with subsequent manual adjustments in MEGA 11 (Tamura et al. 2021). The ITS1 and ITS2 sequences were aligned manually based on their secondary structure. Unalignable positions in the 18S rDNA, ITS and *tuf*A sequences were excluded from the analysis. ModelTest v.3.72 (Posada and Crandall 1998) was utilized to select the optimal evolutionary model, based on hierarchical likelihood ratio tests and the Akaike information criterion. The best-fit model for *tuf*A was GTR+I+G. Due to varying substitution rates amongst the 18S rDNA and ITS rDNA markers, partitioned datasets were analysed separately, with evolutionary models calculated individually for 18S rDNA, ITS1, 5.8S and ITS2. The selected models were: TrN+I+G for 18S rDNA, GTR +G for ITS1, TrNef +I+G for 5.8S and GTR +G for ITS2.

Phylogenetic trees were constructed using both maximum likelihood (ML) and Bayesian methods. ML analysis was conducted with RAxML v.8.1.20 (Stamatakis 2014), with bootstrap support calculated from 1000 replicates. Bayesian analysis was performed using MrBayes v.3.2.6 (Ronquist et al. 2012), running 2 × 10^6^ generations of Markov chain Monte Carlo (MCMC) and sampling every 1000 generations. Stationarity was assumed when the average standard deviation of split frequencies fell below 0.01. The first 25% of trees were discarded as burn-in and the remaining trees were used to construct a consensus tree and estimate posterior probabilities. Final tree visualisation was done using FigTree v.1.4.2 (http://tree.bio.ed.ac.uk/software/figtree/).

### ﻿Compute pairwise distances

These distances were measured using MEGA 11 to calculate pairwise distances within species (within *Gongrosira* and closely-related genera) and between species (within the Stephanosphaerinia clade).

### ﻿Comparison of ITS2 secondary structure

The ITS2 secondary structure was predicted using the Model tool in the ITS2 database (https://its2.bioapps.biozentrum.uni-wuerzburg.de/?group_pub). The original ITS sequence (PQ871574) was annotated and trimmed to obtain the ITS2 sequence using the Annotation tool in the ITS2 database. Multiple sequence alignment and secondary structure alignment were performed using Cluster W (Larkin et al. 2007) in the 4SALE v.1.7 software (Seibel et al. 2008) and CBCs of these secondary structures were calculated. The secondary structure images were edited with Varna v.3.1 (Darty et al. 2009).

## ﻿Results

### ﻿Morphological observations

#### ﻿ Gongrosira leptotricha

Taxon classificationPlantaeChaetophoralesChaetophoraceae

Raineri, 1926

3E94DBC6-EC21-5AA3-B706-F1CF98C48FCA

##### Description.

**Figures 1–4.**
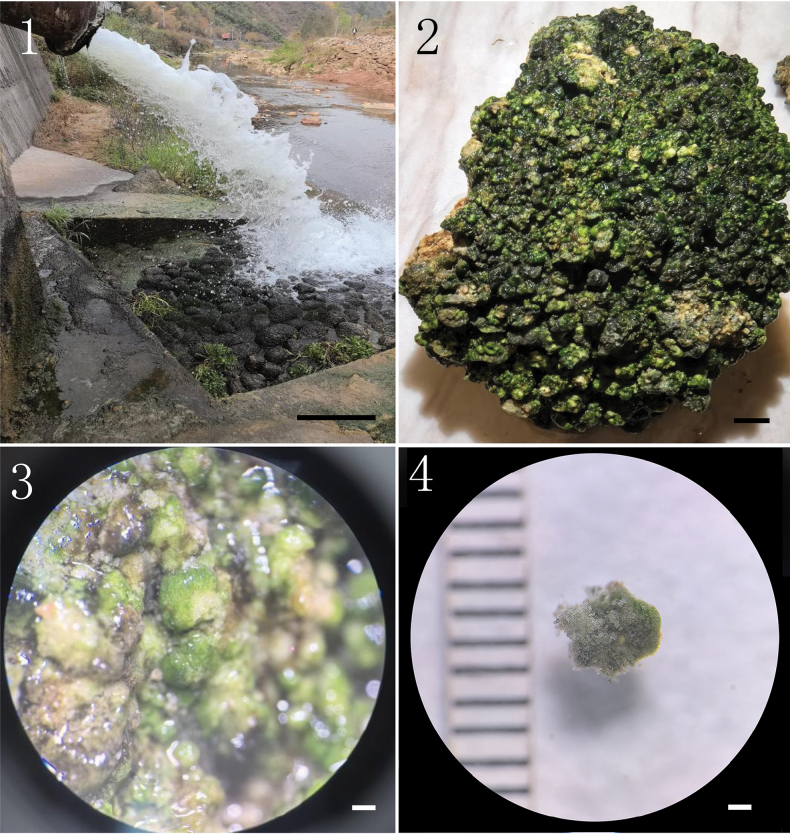
*Gongrosira leptotricha*FACHB-3650: 1. The natural environment of *Gongrosira leptotricha*; 2. Growing on the stones; 3. The outline of the thallus; 4. The size of the thallus. Scale bars 0.5m (1);1 cm (2); 1 mm (3, 4).

**Figures 5–9.**
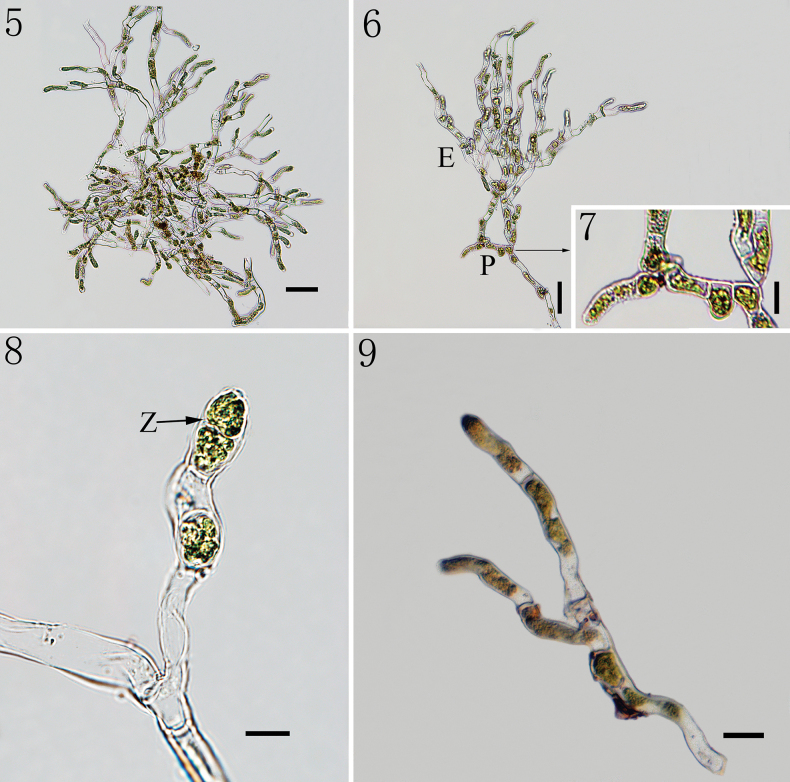
Morphology of *Gongrosira leptotricha*FACHB-3650: 5. Showing fully developed thallus; 6. Showing prostrate and erect system of branched filaments; 7. Showing prostrate part; 8. Showing the upright threads and terminal sporangia; 9. Showing the pseudo-binary branches. (E) erect filaments; (P) prostrate part; (Z) zoosporangia. Figs 5–8: Samples preserved in formalin.Scale bars 50 μm (5–7); 20 μm (8); 10 μm (9).

The thallus is encrusted with loose calcium carbonate crystals, forming dark green spherical, hemispherical or slightly irregular plants on stones in running water (Figs 1–3). The thallus diameter can attain up to 4 mm (Fig. 4). The thallus is composed of prostrate and erect filaments (Figs 5, 6). The prostrate part is creeping, composed of irregularly elliptical or polygonal cells, flattened at the base, which coalesce to form a loose pseudoparenchyma (Fig. 7). The prostrate part extends into short upright threads, with rounded or bottle-shaped terminal sporangia barely larger than the vegetative cells (Fig. 8). The branches are predominantly unilateral, with pseudo-binary branches and the cell wall of the branch is separated from the main axis cell (Fig. 9). Cells of upright threads are irregularly cylindrical or subcylindrical and not constricted or slightly constricted. Cells are 4–7 μm wide, with a length 2–8 times longer than width. The parietal chloroplast contains 1–2 protruding pyrenoids or 3 in some dividing cells (Figs 18–21).

**Figures 10–17.**
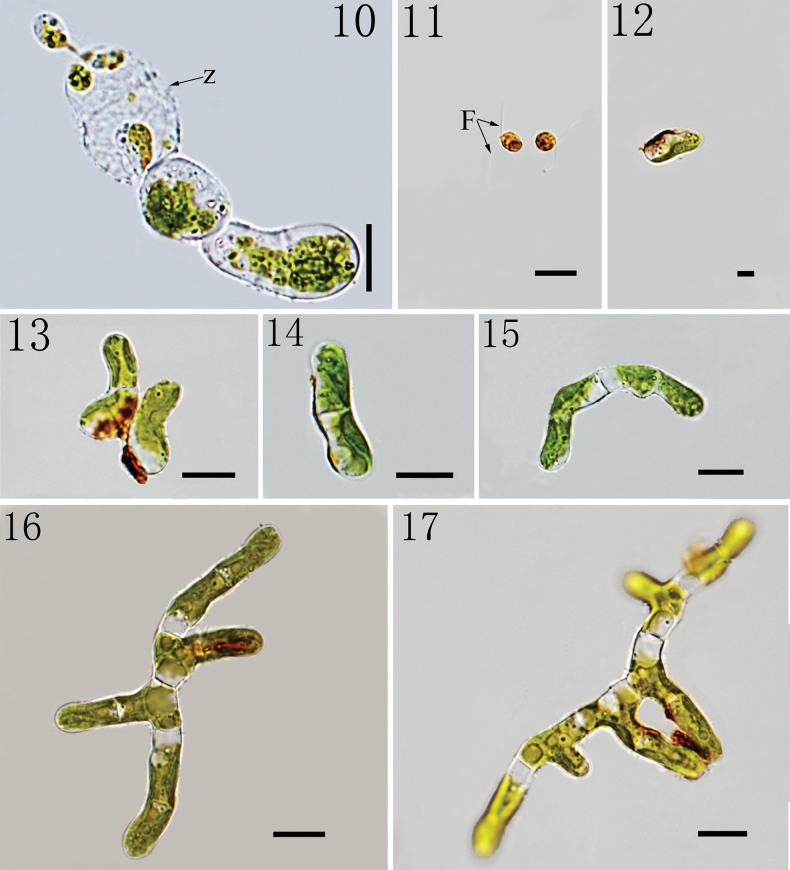
The germination of zoospores and the developmental process of *Gongrosira leptotricha*FACHB-3650: 10. Showing biflagellate zoospores were formed in Terminal sporangia; 11. Zoospores with two flagella (stained with Lugol’s iodine solution); 12, 13. Unidirectional germination of zoospores; 14. Showing the formation of two cells; 15. Filaments elongation; 16, 17. Showing the formation of branches. (Z) zoosporangia; (F) flagella; Scale bars 10 μm (10, 11); 5 μm (12); 10 μm (13–17).

Biflagellate zoospores were formed within terminal sporangia (Fig. 10). Asexual reproduction produces two-flagellated zoospores which are usually elliptic, about 4 × 7 μm (Fig. 11). Zoospores shed flagella, elongate (Figs 12, 13). The germination of zoospores is generally unidirectional to form a filament with two cells (Figs 14, 15). Four-day-old culture, the intercalary cells of the young filament, one-two cells in length, begin to form lateral branches (Fig. 16). The cell wall of branch is separated from the main axis cell (Fig. 17). Each cell is with one parietal chloroplast (Figs 13–17). At early stages of development, the size of the main axis cells and branch cells is not much different, 4–6 μm wide, length 1–2 times longer than width.

Vegetative reproduction occurs via the disintegration of the upright threads into spherical or egg-shaped cells (Figs 18–27), which form two cells through binary fission (Figs 19–21). The cells slowly form short filaments through elongation (Figs 22, 23) and finally develop into a green algal colony with two parts, the prostrate part and the erect part (Figs 24–27).

**Figures 18–27.**
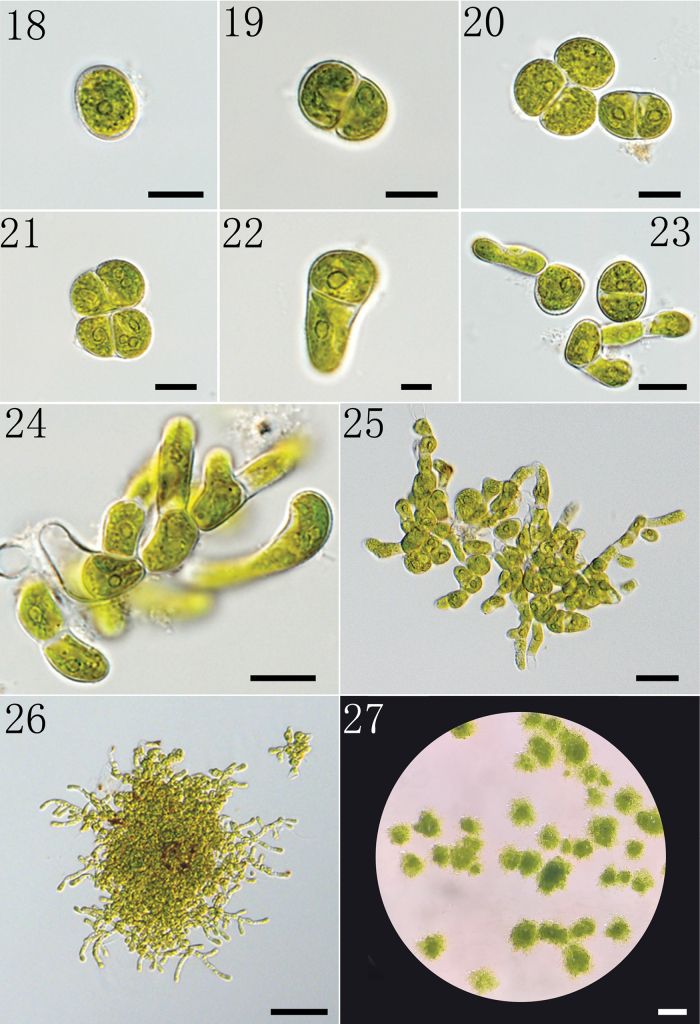
The process of reproduction and development of *Gongrosira leptotricha*FACHB-3650 through binary fission: 18. Showing the single cell; 19. Showing the formation of two cells through binary fission; 20, 21. Showing the germination of multiple cells; 22, 23. Showing the elongation of cells; 24. Showing the branch of thallus; 25. Showing prostrate and erect parts; 26. Showing fully developed thallus; 27. Showing the morphology of thallus on solid medium. Scale bars 10 μm (18–23); 20 μm (24); 10 μm (25); 50 μm (26); 0.5 mm (27).

**Figures 28–31.**
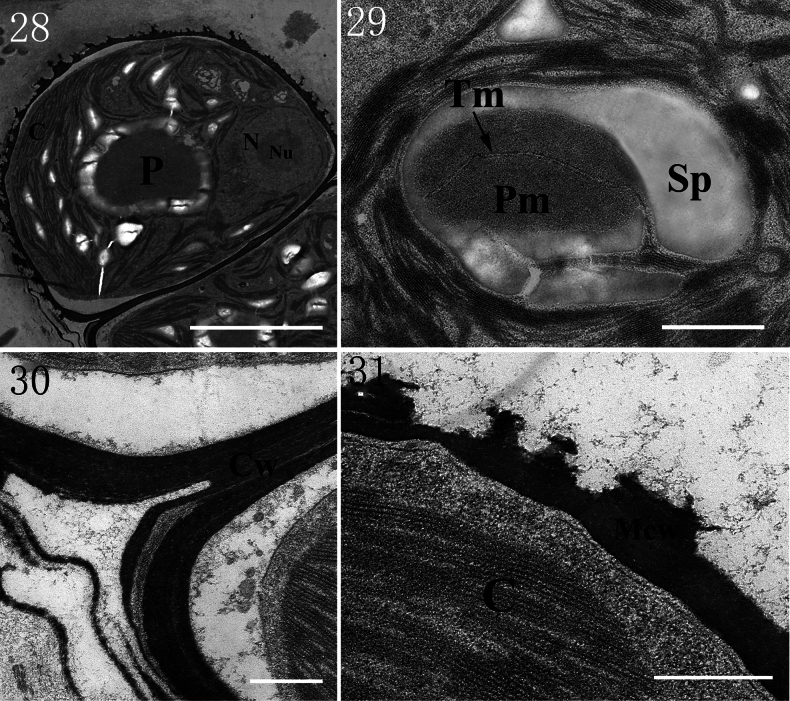
Ultrastructure of *Gongrosira leptotricha*FACHB-3650: 28. Showing the outlet of cell; 29. Pyrenoid with two traversing thylakoid membranes; 30. Cell wall of adjacent cells; 31. Multilayered cell wall of each cell. (C) chloroplast; (Cw) cell wall; (N) nucleus; (Nu) nucleolus; (P) pyrenoid; (Pm) pyrenoid matrix; (Mcw) multi-layered cell wall; (Sp) starch plate; (Tm) thylakoid membranes. Scale bars 5 μm (28); 1 μm (29); 500 nm (30, 31).

Ultrastructural analysis revealed that each pyrenoid is traversed by thylakoid membranes (Figs 28, 29). Each cell has a thick, multilayered cell wall, one nucleus and no plasmodesmata between adjacent cells (Figs 30, 31).

### ﻿Phylogenetic analyses

For the analyses performed in this study, three alignments were generated: (1) the 18S rDNA alignment containing 54 taxa with 1616 bp of the Chlamydomonadales (Chlorophyta); (2) the *tuf*A alignment containing 20 taxa with 816 bp of Chlamydomonadales and (3) concatenated dataset of 16 18S rDNA and ITS rDNA sequences (2396 bp) of the Chlamydomonadales.

The 18S rDNA alignment comprised 1616 sites, of which 455 (28.2%) were variable and 343 (21.2%) were parsimony informative. The concatenated alignment of 16 sequences of 18S rDNA and ITS resulted in 2396 sites, with 807 (33.7%) variable sites and 513 (21.4%) parsimony informative sites. For the *tuf*A alignment, 20 sequences were analysed, yielding 816 sites, of which 415 (50.9%) were variable and 330 (40.4%) were parsimony informative.

**Figure 32.**
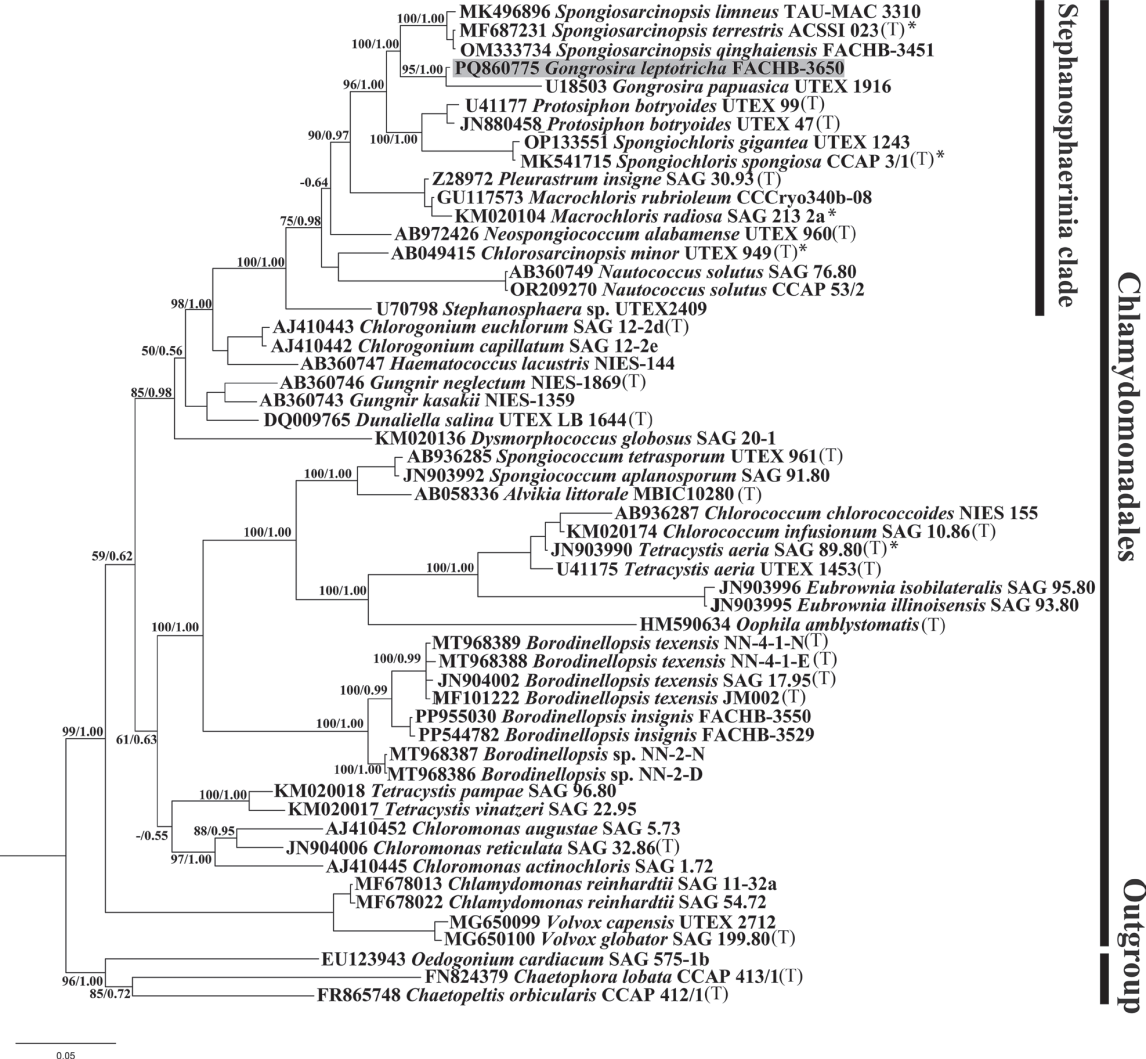
Phylogenetic tree constructed by Bayesian approach, based on 18S rDNA sequences. The numbers above on the nodes represent the Bayesian inference posterior probabilities (PP) and bootstrap support values (BP) from maximum likelihood (ML, constructed by RAxML). Values above 0.5 for BI and 50 for ML are shown. Higher BI values indicate greater reliability of the branch in Bayesian analysis, while higher ML values indicate greater reliability in Maximum Likelihood analysis. The new sequence of this study is shaded in grey. (T) designates type species; asterisks indicate authentic strain.

**Figure 33.**
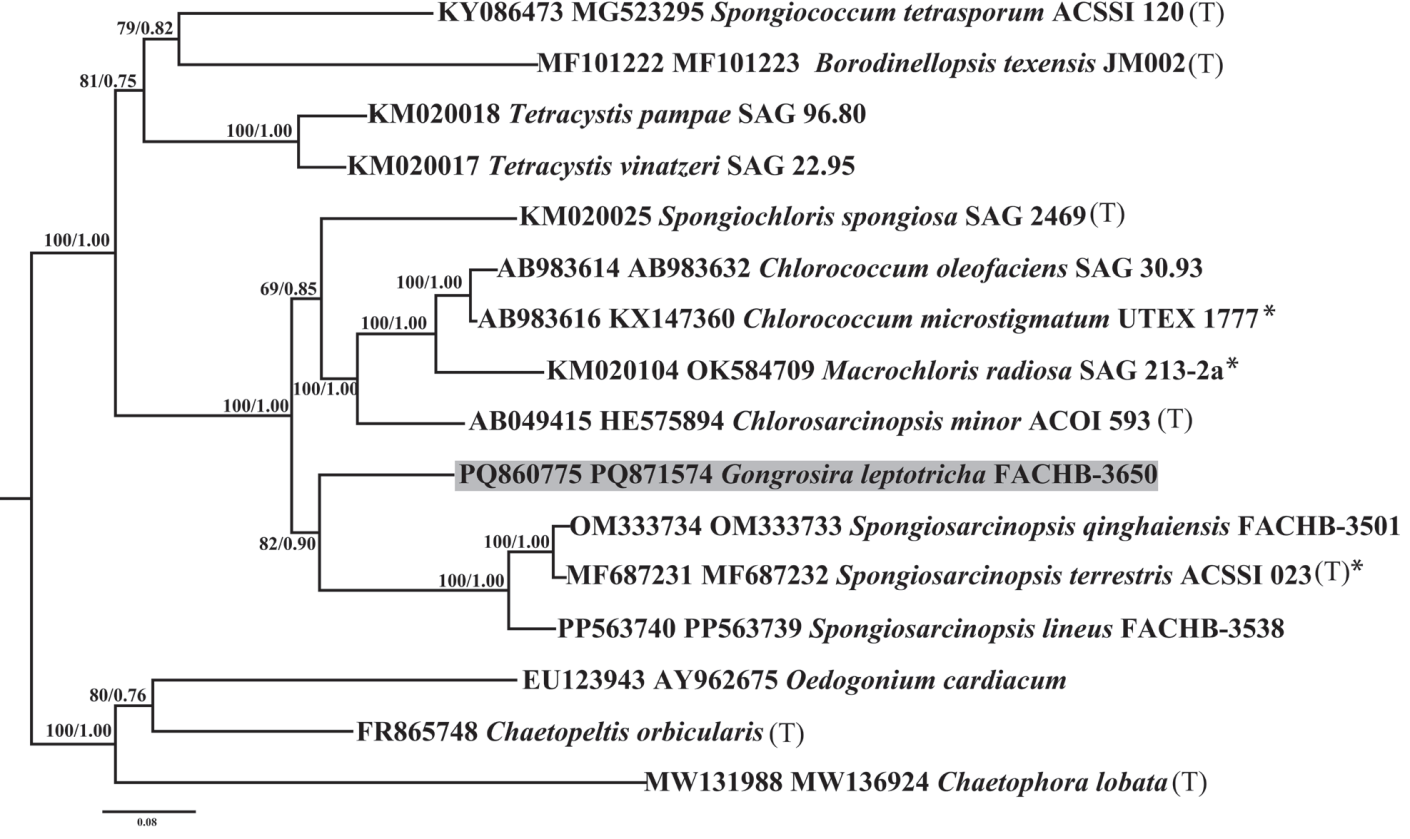
Phylogenetic tree constructed by Bayesian approach, based on 18S rDNA sequences and ITS sequences. The numbers above the nodes represent the Bayesian inference posterior probabilities (PP) and bootstrap support values (BP) from maximum likelihood (ML, constructed by RAxML). Values above 0.5 for BI and 50 for ML are shown. Higher BI values indicate greater reliability of the branch in Bayesian analysis, while higher ML values indicate greater reliability in Maximum Likelihood analysis. The new sequence of this study is shaded in grey. (T) designates type species; asterisks indicate authentic strain.

**Figure 34.**
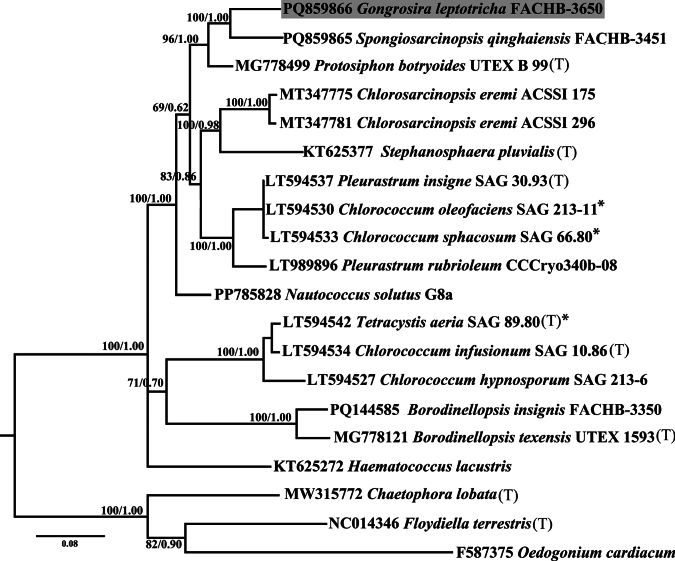
Phylogenetic tree constructed by Bayesian approach, based on *tuf*A sequences. The numbers above the nodes represent the Bayesian inference posterior probabilities (PP) and bootstrap support values (BP) from maximum likelihood (ML, constructed by RAxML). Values above 0.5 for BI and 50 for ML are shown. Higher BI values indicate greater reliability of the branch in Bayesian analysis, while higher ML values indicate greater reliability in Maximum Likelihood analysis. The new sequence of this study is shaded in grey. (T) designates type species; asterisks indicate authentic strain.

Phylogenetic analyses of the 18S rDNA (Fig. 32), *tuf*A (Fig. 34) and concatenated dataset of 18S rDNA and ITS (Fig. 33) sequences were used to determine the unambiguous phylogenetic placement of the *Gongrosira leptotricha* Raineri within the order Chlamydomonadales, Chlorophyceae. Bayesian posterior probabilities (BI values) and Maximum Likelihood bootstrap support values (ML values) are used to assess the reliability of branches. Higher BI values indicate greater reliability of the branch in Bayesian analysis, while higher ML values indicate greater reliability in Maximum Likelihood analysis. Phylogenetic analyses of 18S rDNA, including another species of genus *Gongrosira* (*Gongrosira papuasica* U18503 UTEX 1916), showed that *Gongrosira leptotricha* Raineri is a member of the Stephanosphaerinia clade. *Gongrosira leptotricha* and *Gongrosira papuasica* formed into one clade as the closest relative to the genus *Spongiosarcinopsis* with a high support value (BP/PP, 96/1.00).

The phylogenetic tree of *tuf*A and concatenated sequences of 18S rDNA and ITS showed a similar scenario to that of 18S rDNA, with robust support value (BP/PP, 100/1.00). *Gongrosira leptotricha* Raineri formed a separate clade with the genus *Spongiosarcinopsis*.

### ﻿Pairwise distance computation

The pairwise genetic distances between each species, based on 18S rDNA, were calculated using MEGA11, resulting in Table 2 (partial data). The calculation results show that the genetic distances between *Gongrosira leptotricha* and the closely-related genera *Spongiosarcinopsis*, *Protosiphon* and *Spongiochloris* are much greater than the pairwise distance of 18S rDNA amongst species within those genera.

**Table 2.** Pairwise distances of the 18S rRNA gene of *Gongrosira leptotricha* and closely-related taxa.

Taxon		1	2	3	4	5	6	7	8
1	PQ860775 *Gongrosira leptotricha*	–							
2	U18503 *Gongrosira papuasica*	0.0193174501	–						
3	MK496896 *Spongiosarcinopsis limneus*	0.0175557056	0.0329824561	–					
4	MF687231 *Spongiosarcinopsis terrestris*	0.0204207921	0.0341274952	0.0020256583	–				
5	OM333734 *Spongiosarcinopsis qinghaiensis*	0.0204207921	0.0341274952	0.0020256583	0.0000000000	–			
6	U41177 *Protosiphon botryoides*	0.0266418835	0.0354609929	0.0229885057	0.0247831475	0.0247831475	–		
7	JN880458 *Protosiphon botryoides*	0.0253712871	0.0341274952	0.0202565834	0.0235148515	0.0235148515	0.0037174721	–	
8	OP133551 *Spongiochloris gigantea*	0.0340346535	0.0431423052	0.0270087779	0.0327970297	0.0327970297	0.0272614622	0.0259900990	–
9	MK541715 *Spongiochloris spongiosa*	0.0327970297	0.0418544752	0.0256583390	0.0327970297	0.0327970297	0.0260223048	0.0247524752	0.0012376238

### ﻿ITS2 secondary structure

The ITS2 secondary structure was annotated and the ITS2 sequence of *Gongrosira leptotricha* with a length of 282 bp was folded into its secondary structure. Multiple sequence alignment and secondary structure alignment were performed amongst the secondary structures of *Gongrosira leptotricha* (PQ871574), *Spongiosarcinopsis terrestris* (MF687232) and *Spongiosarcinopsis qinghaiensis* (OM333733) and CBCs were calculated for these structures, resulting in Suppl. materials 2, 3. When comparing *Gongrosira leptotricha* with *Spongiosarcinopsis terrestris* (Suppl. material 2), the lengths of their ITS2 sequences were 282 bp and 263 bp, respectively and their secondary structures exhibited five CBCs and seven hemi-compensatory base changes (h-CBCs). When comparing *Gongrosira leptotricha* with *Spongiosarcinopsis qinghaiensis* (Suppl. material 3), the lengths of their ITS2 sequences were 282 bp and 264 bp, respectively and their secondary structures exhibited five CBCs and six hemi-compensatory base changes (h-CBCs).

## ﻿Discussion

Members of the genus *Gongrosira* are differentiated by a range of morphological traits, including cell size, the extent of development in erect and prostrate system, branching patterns, sporangia placement and the occurrence of calcification (Printz 1964; Liu et al. 2024). Notably, *Gongrosira leptotricha* Raineri exhibits distinct features such as terminal sporangia that are only marginally larger than vegetative cells, reduced cell dimensions, an elongated cell shape with a high length-to-width ratio, limited branching and a specific number of pyrenoids per cell (Chodat et al. 1926; Printz 1964). The diagnostic features of all *Gongrosira* species are shown in Suppl. material 1. All previous records report the *Gongrosira leptotricha* Raineri occurring on stones in rivers (Chodat et al. 1926; Täuscher 2016; Doege et al. 2022). Samples in this study agreed well with the description by Chodat et al. (1926) and Printz (1964) and were identified as *Gongrosira leptotricha* Raineri. This is the first recorded instance of this alga being found in China. The slight differences in cell size might be attributed to incomplete development or phenotypic plasticity (Liu et al. 2019a; 2024). Encrustation is not considered a good taxonomic character. The presence or absence of lime encrustation may be related to the growth environment (Pentecost 1988). The crusts associated with *Gongrosira* may not be a direct result of the alga’s metabolic activities, but rather indicative of the chemical composition of the water in which it thrives (Johnson and John 1992).

Ultrastructural analysis challenges the classification of *Gongrosira leptotricha* Raineri within the Chaetophorales. In Chaetophorales, cells are interconnected by plasmodesmata (Stewart et al. 1973; John 1984; Melkonian 1990); however, *Gongrosira leptotricha* Raineri lacks plasmodesmata, supporting its exclusion from this order. A comparable case is observed in *G. papuasica* (Borzì) Tupa (Watanabe et al. 1992), which further supports the exclusion of *G. papuasica* (Borzì) Tupa from the Chaetophorales, a conclusion corroborated by molecular data. Two sequences of *G. papuasica* (Borzì) Tupa UTEX 1916 (accessions U18503 and DQ015756) deposited in GenBank are classified within Chlorophyceae. Notably, detailed morphological studies of *G. papuasica* (Borzì) Tupa UTEX 1916 have been conducted by Tupa (1974) and Johnson and John (1992). Ultrastructural studies of *Gongrosira papuasica* (Borzì) Tupa and *Gongrosira leptotricha* Raineri revealed that the pyrenoid is traversed by thylakoid membranes (Watanabe et al. 1992 and herein), suggesting that they are closely related. The pyrenoid structures of *Gongrosira leptotricha* Raineri closely resemble those of *G. papuasica* (Borzì) Tupa and differ from those in Chaetophorales, where thylakoid bands are either appressed to the pyrenoid matrix periphery or penetrate only a short distance into the matrix (Stewart et al. 1973; Liu et al. 2019b).

Phylogenetic analyses indicate that many species of the genus *Gongrosira*, previously classified within the order Chaetophorales (Chlorophyceae), actually belong to the Ulvophyceae (Liu et al. 2019a; 2024). In this study, we provide the first molecular record of *Gongrosira leptotricha* Raineri. Molecular data, including 18S rDNA, ITS and chloroplast-encoded *tuf*A, strongly support that *Gongrosira leptotricha* Raineri is a member of the Chlamydomonadales (Chlorophyceae) rather than the Chaetophorales (Chlorophyceae). *Gongrosira leptotricha* Raineri shows the closest relationship with *G. papuasica* (Borzì) Tupa, forming a sister clade with the genus *Spongiosarcinopsis*. The moderate to low support values for this relationship may be attributed to the limited number of samples and DNA sequences available at the time. Recently, several new members of the genus *Spongiosarcinopsis* have been described (Temraleeva et al. 2018; Lortou et al. 2022; Yan et al. 2022), suggesting that the diversity of the Stephanosphaerinia clade is underestimated.

Notably, *Gongrosira leptotricha* Raineri and *G. papuasica* (Borzì) Tupa exhibit distinct morphological and ecological traits, such as freshwater habitat, parietal chloroplasts and filamentous thalli, which contrast sharply with the soil-dwelling, spongy or net chloroplasts, unicellular and spherical thalli or forming cell packets of *Spongiosarcinopsis* species. In terms of the comparative ultrastructural analysis, the number of pyrenoids is also different. These differences are further supported by molecular data. The pronounced difference appears in the 18S rRNA gene sequences of *Gongrosira leptotricha* Raineri compared to *Spongiosarcinopsis* species, showing a 1.9% mean pairwise genetic distance significantly greater than the 0–0.2% divergence observed amongst *Spongiosarcinopsis* species. Based on the evidence presented, *Gongrosira leptotricha* Raineri and *G. papuasica* (Borzì) Tupa should not be assigned to any currently known genus, but, instead, form a separate clade within the Chlamydomonadales. It is acknowledged that the phylogenetic positions of many species within the genus *Gongrosira* Kützing remain unresolved, particularly that of the type species *Gongrosira sclerococcus* Kützing, meaning the true phylogenetic position of the genus *Gongrosira* is still uncertain. Despite the significant differences observed in both morphological and molecular data, we have refrained from drawing definitive conclusions regarding the necessity of proposing a new genus for the clade comprising *Gongrosira leptotricha* and *G. papuasica*, primarily due to the limited number of available sequences and specimens. Therefore, we propose to temporarily classify *Gongrosira leptotricha* and *G. papuasica* as a distinct clade within the Chlamydomonadales, retaining their original names.

So far, phylogenetic positions of four species of the genus *Gongrosira* have been well confirmed. *Gongrosira burmanica* Skuja and *Gongrosira fluminensis* Fritsch have both been reclassified under the Ulvales (Ulvophyceae) (Liu et al. 2019a; 2024), while *Gongrosira leptotricha* Raineri and *G. papuasica* (Borzì) Tupa have been transferred to the Chlamydomonadales (Chlorophyceae) (as demonstrated in this study). These findings may contribute to resolving the taxonomic complexities surrounding the genus *Gongrosira* Kützing. While the true phylogenetic position of the genus *Gongrosira* Kützing remains uncertain, it is clear that the broadly defined genus *Gongrosira*, which encompasses multiple morphologically similar groups, requires division into smaller, more precise taxonomic units.

Future comprehensive investigations involving additional specimens, combined with natural morphological observations, culture-based studies and molecular analyses, will be essential to re-assess the microfilamentous genus *Gongrosira* and uncover its hidden diversity within the Ulvophyceae and Chlorophyceae. These studies should prioritise expanding the molecular dataset for additional *Gongrosira* species, particularly the type species *Gongrosira sclerococcus*, to further resolve the taxonomic uncertainties within this genus.

## ﻿Conclusions

The polyphasic taxonomic analysis indicates that *Gongrosira leptotricha* Raineri should be excluded from the order Chaetophorales and recognised as a new member of the Stephanosphaerinia clade within the Chlamydomonadales (Chlorophyceae). *Gongrosira leptotricha* and *Gongrosira papuasica* form a distinct lineage that does not align with any currently-recognised genus. The name *Gongrosira leptotricha* has been retained due to the limited availability of sequences and specimens. Further comprehensive research, particularly focusing on the type species *Gongrosira sclerococcus*, is crucial to refine the classification framework and resolve the taxonomic uncertainties within this genus.

## Supplementary Material

XML Treatment for Gongrosira leptotricha

## ﻿Additional information

### Use of AI

No use of AI was reported.

### Author ORCIDs

Qiumei Lan https://orcid.org/0009-0004-5135-8763

### Data availability

All of the data that support the findings of this study are available in the main text or Supplementary Information.

## Supplementary materials

Supplementary material 1 Table of diagnostic features for all *Gongrosira* species Data type docx https://binary.pensoft.net/file/1410415This dataset is made available under the Open Database License (http://opendatacommons.org/licenses/odbl/1.0/). The Open Database License (ODbL) is a license agreement intended to allow users to freely share, modify, and use this Dataset while maintaining this same freedom for others, provided that the original source and author(s) are credited.Qiumei Lan, Qingyu Dai, Huan Zhu, Guoxiang Liu, Benwen Liu

Supplementary material 2 The ITS2 secondary structure between *Gongrosira leptotricha*FACHB-3650 and *Spongiosarcinopsis terrestris* Data type tif Explanation note The darker the colour, the more significant the variation. Differences between the two species have been indicated by arrows in the figure, red for CBCs and blue for h-CBCs. https://binary.pensoft.net/file/1410416This dataset is made available under the Open Database License (http://opendatacommons.org/licenses/odbl/1.0/). The Open Database License (ODbL) is a license agreement intended to allow users to freely share, modify, and use this Dataset while maintaining this same freedom for others, provided that the original source and author(s) are credited.Qiumei Lan, Qingyu Dai, Huan Zhu, Guoxiang Liu, Benwen Liu

Supplementary material 3 The ITS2 secondary structure between *Gongrosira leptotricha*FACHB-3650 and *Spongiosarcinopsis qinghaiensis* Data type tif Explanation note The darker the colour, the more significant the variation. Differences between the two species have been indicated by arrows in the figure, red for CBCs and blue for h-CBCs. https://binary.pensoft.net/file/1410417This dataset is made available under the Open Database License (http://opendatacommons.org/licenses/odbl/1.0/). The Open Database License (ODbL) is a license agreement intended to allow users to freely share, modify, and use this Dataset while maintaining this same freedom for others, provided that the original source and author(s) are credited.Qiumei Lan, Qingyu Dai, Huan Zhu, Guoxiang Liu, Benwen Liu
