# Supplementary material for: ﻿Re-assignment of Gongrosira leptotricha, a newly-recorded species in China, to Stephanosphaerinia clade (Chlamydomonadales, Chlorophyceae): insights from morphological and phylogenetic analyses
Source: PhytoKeys. 2025 Sep 5;262:171–89. doi: 10.3897/phytokeys.262.152528 (PMC12432526; doi:10.3897/phytokeys.262.152528)
Supplement: Supplementary material 1 — Table of diagnostic features for all Gongrosira species [file phytokeys-262-171_article-152528__-s001.docx]

Table S1. Table of diagnostic features for all *Gongrosira* species.

| Species Name | Sporangien Position and Size | Thallus | Base | Erect filaments |
| --- | --- | --- | --- | --- |
| *G. debaryana* Rabenhorst | Terminal, much larger than vegetative cells | Crustose, few-branched water filaments | Pseudoparenchymatous disc | 15-30 μm thick, few-celled, very short, scarcely branched |
| *G. stagnalis* (G.S.West) Schmidle | Terminal, much larger than vegetative cells | Crustose, few-branched water filaments | Pseudoparenchymatous disc | 15-30 μm thick, water filaments up to 500 μm long, few and short branches |
| *G. fluminensis*^✳^ F.E*.*Fritsch | Terminal, much larger than vegetative cells | Crustose, few-branched water filaments | Pseudoparenchymatous disc | About 7 μm thick |
| *G. lacustris* Brand | Terminal, much larger than vegetative cells | Crustose, few-branched water filaments | Filamentous cells penetrating the substrate, cells up to 14 μm thick | Filamentous cells penetrating the substrate, cells up to 14 μm thick |
| *G. prostrata* Jao | Terminal, much larger than vegetative cells | Cushion-shaped, water filaments highly branched | - | Rare, single-celled |
| *G. pygmaea* Kützing | Terminal, much larger than vegetative cells | Cushion-shaped, water filaments highly branched | - | 15-20 μm thick, highly branched |
| *G. sclerococcus* Kützing, nom. Illeg. = *G*. *viridis* (Kirzing) De Toni | Terminal, much larger than vegetative cells | Cushion-shaped, water filaments highly branched | - | 10 μm thick, highly branched, branching in all directions |
| *G. recurvata* (Wittrock & Nordstedt) Printz | Terminal, much larger than vegetative cells | Cushion-shaped, water filaments highly branched | - | 8-14 μm thick, upper branching unilateral |
| *G. trentepohliopsis* Schmidle | Terminal, much larger than vegetative cells | Cushion-shaped, water filaments highly branched | - | 6-8 μm thick |
| *G. circinnata* (Borzì) Schmidle | Terminal, slightly larger than vegetative cells | Upper cells of water filaments chlorophyll-green | - | 10-15 μm thick, branches recurved, comb-like branching |
| *G. fastigiata* (Borzì) Schmidle | Terminal, slightly larger than vegetative cells | Upper cells of water filaments chlorophyll-green | - | 10-15 μm thick, branches erect, branching equal in height |
| *G. disciformis* F.E.Fritsch | Terminal, slightly larger than vegetative cells | Basal part consists of highly branched, creeping, parenchymatous filaments | - | Cells 1-2 times longer than wide |
| *G. burmanica*^✳^ Skuja | Terminal, slightly larger than vegetative cells | Basal part consists of radially radiating, pseudodichotomously branched filaments | - | Cells 1-2 times longer than wide |
| *G. calcifera* Willi Krieger | Terminal, slightly larger than vegetative cells | Basal part consists of highly branched, creeping, parenchymatous filaments | - | filaments Cells 2-3(-4) times longer than wide |
| *G. incrustans* (Reinsch) Schmidle | Terminal, slightly larger than vegetative cells | Cells 6-10 μm thick | - | - |
| *G. leptotricha* Raineri | Terminal, slightly larger than vegetative cells | Cells 4-6 μm thick | - | - |
| *G. schmidlei* P.G.Richter | Terminal, slightly larger than vegetative cells | Only the terminal cell contains chlorophyll | - | - |
| *G. scourfieldii* G.S.West | Intercalary | - | - | Cells up to 9 times longer than wide |
| *G. calcarea* (Nayal) Printz | Intercalary | Calcified, branching unilateral | - | Cells 1-2 times longer than wide |
| *G. tibetana* (Skuja) Printz | Intercalary | Non-calcified, branching opposite | - | Cells 1-2 times longer than wide |

(✳) indicates species transferred to other genera; (-) data not available.
