# Supplementary figures and images for: ﻿Re-assignment of Gongrosira leptotricha, a newly-recorded species in China, to Stephanosphaerinia clade (Chlamydomonadales, Chlorophyceae): insights from morphological and phylogenetic analyses
Source: PhytoKeys. 2025 Sep 5;262:171–89. doi: 10.3897/phytokeys.262.152528 (PMC12432526; doi:10.3897/phytokeys.262.152528)

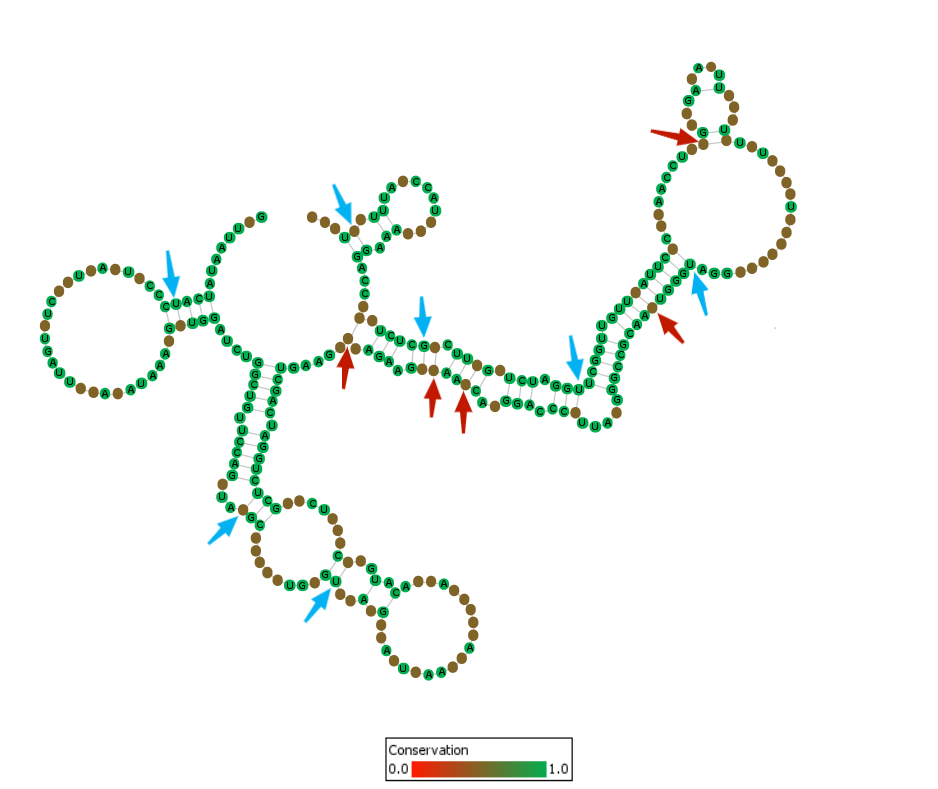

Supplement: Supplementary material 2 — The ITS2 secondary structure between Gongrosira leptotrichaFACHB-3650 and Spongiosarcinopsis terrestris [file phytokeys-262-171_article-152528__-s002.tif]

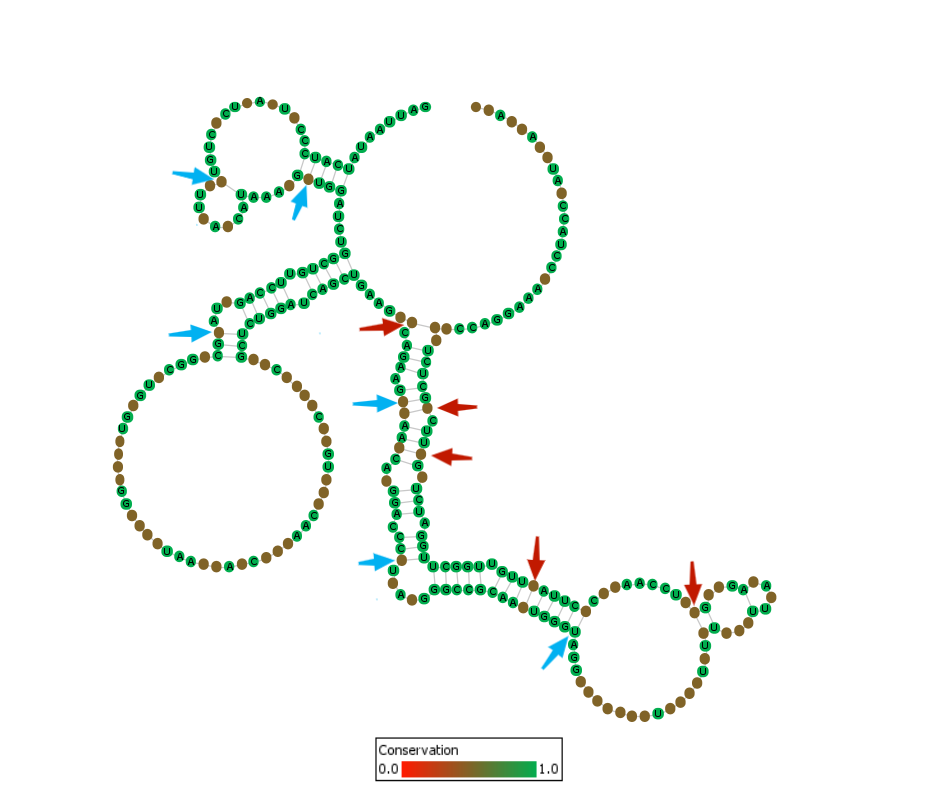

Supplement: Supplementary material 3 — The ITS2 secondary structure between Gongrosira leptotrichaFACHB-3650 and Spongiosarcinopsis qinghaiensis [file phytokeys-262-171_article-152528__-s003.tif]
